# A new approach to diagnosing and researching developmental prosopagnosia: Excluded cases are impaired too

**DOI:** 10.3758/s13428-022-02017-w

**Published:** 2022-12-02

**Authors:** Edwin J. Burns, Elizabeth Gaunt, Betiel Kidane, Lucy Hunter, Jaylea Pulford

**Affiliations:** Department of Psychology, Edge Hill University, Ormskirk, UK

**Keywords:** Diagnosing, Criteria, Prosopamnesia, Prosopagnosia, Single-case analysis, Apperceptive, Associative

## Abstract

Developmental prosopagnosia is characterized by severe, lifelong difficulties when recognizing facial identity. Unfortunately, the most common diagnostic assessment (Cambridge Face Memory Test) misses 50–65% of individuals who believe that they have this condition. This results in such excluded cases’ absence from scientific knowledge, effect sizes of impairment potentially overestimated, treatment efficacy underrated, and may elicit in them a negative experience of research. To estimate their symptomology and group-level impairments in face processing, we recruited a large cohort who believes that they have prosopagnosia. Matching prior reports, 56% did not meet criteria on the Cambridge Face Memory Test. However, the severity of their prosopagnosia symptoms and holistic perception deficits were comparable to those who did meet criteria. Excluded cases also exhibited face perception and memory impairments that were roughly one standard deviation below neurotypical norms, indicating the presence of objective problems. As the prosopagnosia index correctly classified virtually every case, we propose it should be the primary method for providing a diagnosis, prior to subtype categorization. We present researchers with a plan on how they can analyze these excluded prosopagnosia cases in their future work without negatively impacting their traditional findings. We anticipate such inclusion will enhance scientific knowledge, more accurately estimate effect sizes of impairments and treatments, and identify commonalities and distinctions between these different forms of prosopagnosia. Owing to their atypicalities in visual perception, we recommend that the prosopagnosia index should be used to screen out potential prosopagnosia cases from broader vision research.

## Introduction

Developmental prosopagnosia (DP) is a lifelong disorder characterized by severe difficulties when recognizing facial identity (De Hann, 1999; Duchaine, 2000; Kress & Daum, 2003; McConachie, 1976; Nunn et al., 2001). Its prevalence is commonly reported to be 2–3% (Kennerknecht et al., 2006; Kennerknecht et al., 2008), although one study has suggested this could be as high as 5% (Bennetts et al., 2017). It is common for these individuals to suffer difficulties with other aspects of cognition, including facial emotion processing (Biotti & Cook, 2016; Burns et al., 2017c; Dinkelacker et al., 2011; Palermo et al., 2011; Tsantani et al., 2022a, 2022b), body perception (Biotti et al., 2017a; Righart & de Gelder, 2007), reading (Burns & Bukach, 2021; Burns & Bukach, 2022) and object recognition (Burns et al., 2019; Geskin & Behrmann, 2018). While researchers are unsure if DP is caused by genetic atypicalities and/or environmental disruptions (Susilo & Duchaine, 2013), it is associated with neural abnormalities in face-related brain regions (Avidan & Behrmann, 2009; Furl et al., 2011; Garrido et al., 2009; Jiahui et al., 2018; Rivolta et al., 2014; Song et al., 2015; Thomas et al., 2008; Towler et al., 2017; Towler et al., 2016; Van den Stock et al., 2008; Zhang et al., 2015; Zhao et al., 2018), which can be predictive of their difficulties with faces (Furl et al., 2011).

Despite research into prosopagnosia growing rapidly over the last 10 – 20 years, there are still no universally agreed upon methods to diagnose the condition. Instead, researchers rely upon a small number of face processing tests to determine a diagnosis. The most common of these is the Cambridge Face Memory Test (CFMT: Duchaine & Nakayama, 2006; Arrington et al., 2022), where participants study a series of unfamiliar targets before picking them out of a line-up containing unstudied lures. To illustrate researchers’ heavy reliance upon the CFMT, we reviewed all 15 DP papers published in 2020 (Table 1) and found 14 required their DP cases to exhibit some form of impairment, with the most common being severe: i.e., greater than – 2 SDs relative to a control mean (nine papers). This cut-off does vary marginally between studies, but it is close to employing a single-case analysis approach (e.g., the Crawford’s *t* test, Crawford & Howell, 1998) for detecting impairment. In addition to the CFMT, researchers have commonly assessed prosopagnosia through two other measures: a famous faces test (FFT, eleven studies), where images of celebrities need to be identified, and the Cambridge Face Perception Test (CFPT, eight papers, Duchaine et al., 2007b), where a target face and a distractor face are morphed together in varying degrees of influence, creating a range of new faces that need to be ordered in similarity to the target.

**Table 1:** The fifteen developmental prosopagnosia papers published in 2020 and their inclusion criteria for diagnosing the condition. Fourteen required some form of impairment on the Cambridge Face Memory Test (CFMT: nine papers mentioned > – 2 SDs poorer than the control mean, which equates to < 43 trials correct) illustrating researchers’ heavy reliance upon this measure for a diagnosis. Other assessments included a prosopagnosia symptom questionnaire (prosopagnosia index: PI20; Shah et al., 2015), Cambridge Face Perception Test (CFPT; Duchaine et al., 2007b) and Famous Faces Test (FFT)

Paper	PI20	CFMT	CFPT	FFT	Notes
(Adams et al. 2020)	NA	> – 2 SDs	> – 2 SDs	> – 2 SDs	Must be impaired on two of the CFMT, CFPT and FFT
(Bylemans et al. 2020)	Screening, not specified	≤ 46 trials correct	≥ 50 errors	NA	Must be impaired on both CFMT and CFPT
(Djouab et al. 2020)	Screening	>– 2 SDs	NA	>– 2 SDs	Must be impaired on CFMT and another test (e.g., old/new)
(Fisher et al. 2020)	≥ 65	< 45 trials correct	No criteria	>– 2 SDs	Report in text all > – 2 SDs on CFMT and FFT (although two in table do not meet this criteria)
(Fry et al. 2020)	> 64	≤ 44 trials correct	NA	>– 2 SDs	All three impairments were required
(Gerlach et al. 2020)	NA	≤ 46 trials correct	No Criteria	NA	Only impairment on CFMT
(Jiahui et al. 2020)	NA	>– 2 SDs	NA	>– 2 SDs	All impaired on two of CFMT, FFT, or an old/new test. One borderline CFMT case (*z* = – 1.9) included
(Mishra et al. 2020)	> 65	< 44 trials correct	NA	< 70%	Must reach criteria on all three
(Murray and Bate, 2020)	NA	< 42 trials correct	No Criteria	No Criteria	Assessed test–retest reliabilities, so no initial cut-offs
(Pertzov et al. 2020)	NA	> – 2 (non-age matched) and > – 1.3 (age matched)	NA	>2 SDs	Must meet all criteria
(Stumps et al. 2020)	> 65	> – 1SD (mild) > – 2 SDs (major)	No criteria	> – 1SD (mild) > – 2 SDs (major)	Must meet CFMT and FFT criteria
(Tian et al. 2020)	NA	NA	NA	No Criteria	Variety of screenings, but must score > – 1 SD on old/new
(Tsantani & Cook, 2020)	> – 2 SDs	> – 2 SDs	NA	NA	All met CFMT and PI20 impairment
(Tsantani et al. 2020)	> – 2Ds	> – 2 SDs	No Criteria	NA	Impairment on PI20 and CFMT
(Wilcockson et al. 2020)	NA	>– 2 SDs	No Criteria	>– 2 SDs	Must meet CFMT and FFT criteria

In an ideal world, requiring objective impairments on a face processing task would seem a reasonable criterion for a diagnosis. This is because in the traditional neuropsychological approach for detecting deficits, scoring more than 1.96 SDs below a control mean on a cognitive task (i.e., bottom 2.5% of the general population, although this cut-off is commonly rounded up to 2 SDs) is the two-tailed threshold for determining a patient is significantly abnormal (McIntosh & Rittmo, 2021). Such requirements reassure researchers that these individuals are functioning abnormally in a specific domain, given the unlikely chance that a neurotypical individual would perform so poorly (Young et al., 1993).

### Introducing the problems with excluded developmental prosopagnosia)

However, this assumption has many pitfalls when it comes to diagnosing developmental prosopagnosia. The first is that roughly 50–65% of those who believe that they have this condition fail to score more than 2 SDs below the neurotypical CFMT mean (Bate et al., 2019a; Burns et al., 2014a; Murray & Bate, 2020; Ulrich et al., 2014), with similar misses apparent when attempting to diagnose DP using the FFT (25% missed, Bate et al., 2019a) and CFPT (80% missed, Bate et al., 2019a). These issues have historical precedence, with one of the earliest papers noting a self-identified case that failed to perform abnormally poorly on face processing tasks (De Haan, 1999). In our experience, these cases appear indistinguishable when interviewed from those who do meet criteria: they commonly fail to recognize people personally known to them, they mix up characters when watching TV shows or films, and they try to recognize people by distinctive features, such as a large nose or bushy eyebrows. Yet when it comes to assessing their complaints objectively, we are unable to, at least from the perspective of single-case criteria, confirm a diagnosis^1^ (Bate et al., 2019a; Burns et al., 2014a; De Haan, 1999; Murray & Bate, 2020).

So far, researchers have suggested that these individuals do not suffer from prosopagnosia, but may simply have a failure in meta-cognition (Arizpe et al., 2019; Bate et al. 2019a), i.e., they are unable to accurately judge that their face recognition skills appear perfectly normal, at least when assessed on tasks such as the CFMT and FFT. Here, we refer to these individuals as *Excluded* developmental prosopagnosia, because when they fail to meet the criteria for a diagnosis they are excluded from research, resulting in their almost complete absence from the scientific literature. By contrast, we refer to those cases who do meet criteria as *Classical* developmental prosopagnosia, since traditionally, they are the classic cases who have met criteria for a diagnosis and thus been reported on. In the subsequent sections, we outline many of the issues that will afflict the developmental prosopagnosia field if we continue to exclude over 50% of potential cases from our research. At times, we will use the CFMT to illustrate these problems to the reader, but similar troubles are apparent for the other two most common cognitive assessments, i.e., the CFPT and FFT.

### The single-case approach may have low power for diagnosing DP

First, we do not know what the baseline level of impairment those with DP as a group suffer from when assessed on tests such as the CFMT, CFPT and FFT. Right now, researchers are using conservative cut-offs to diagnose prosopagnosia (i.e., > – 2SD impairment below the control mean; Adams et al., 2020; Djouab et al., 2020; Jiahui et al., 2020; Tsantani & Cook, 2020; Tsantani et al., 2020), but what if excluded DP cases do have insights into their face-recognition difficulties when they complain that they are unable to identify friends and family members (Livingston & Shah, 2018)? Surely it is quite easy to tell if you are failing to recognize important people in your life on a regular basis? What if their ability to self-identify as having prosopagnosia is entirely accurate, but our objective tasks and cut-offs are imperfect at capturing these difficulties? This would mean that researchers are forcing a diagnostic criterion (i.e., significantly impaired on the CFMT at the single-case level) upon a population that may seem mostly unimpaired when using this approach.

**Fig. 1:**
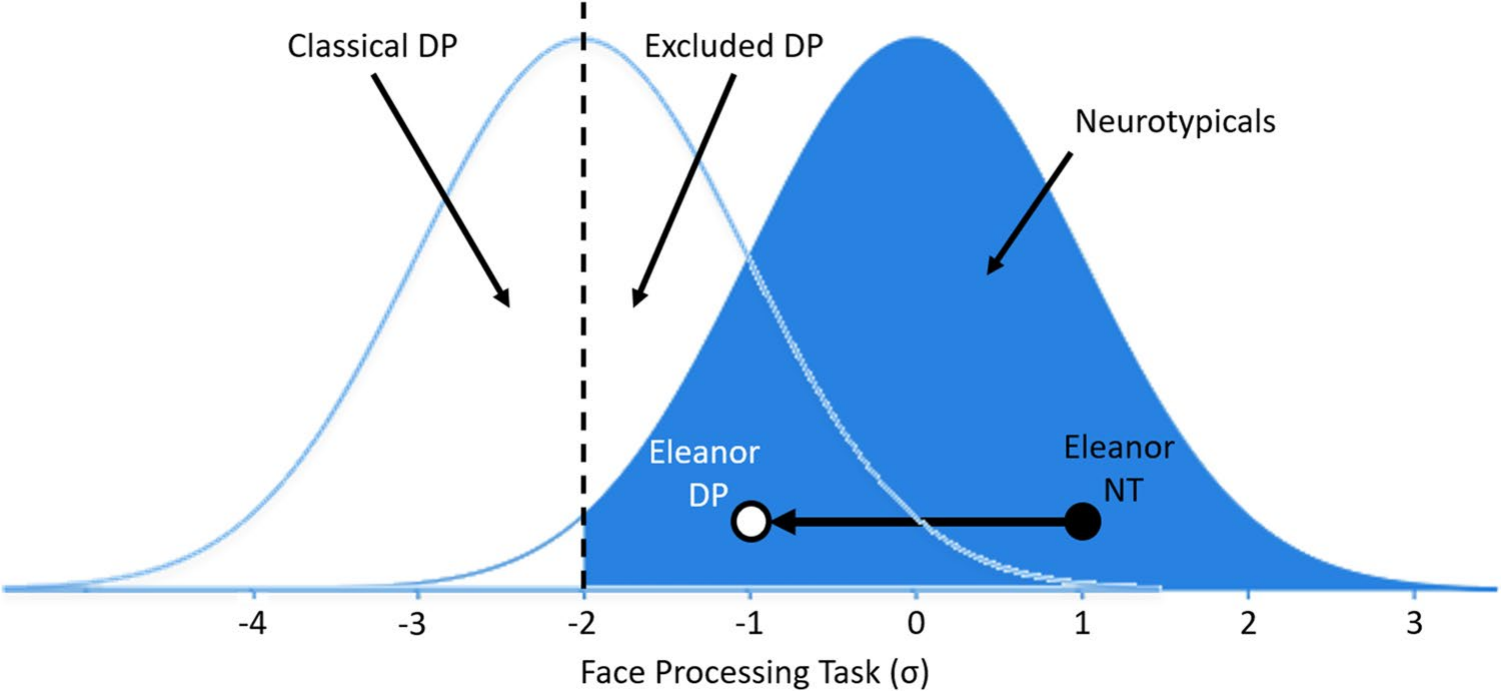
Illustration of potentially missed DP cases when using the single-case approach. The *blue distribution on the right* represents the neurotypical population on a hypothetical face processing task. Researchers will typically diagnose those who score in the white tail-end of this neurotypical distribution (i.e., those impaired more than – 2 SDs below the control mean, *dashed vertical line*). However, if DP cases are a distinct normally distributed group on this task (*white Gaussian distribution on the left*), with their mean level of impairment landing – 2 SDs below the control mean, then half of all cases will fail to meet criteria for a diagnosis when using this approach (i.e., the top half of this distribution scoring above the dashed vertical cut-off will be excluded). To illustrate this further, imagine neurotypical Eleanor (NT *black circle*) scores above average (i.e., 1 SD) on our face processing task, but we then induce in her the developmental disruption that causes DP. Eleanor the DP (*white circle*) will suffer from severe difficulties in daily life when recognizing faces, score within the DP population’s white distribution, but the single-case approach will fail to recognize her as suffering from prosopagnosia. Despite the severe – 2 SD impairment Eleanor has suffered, she will never meet the criteria required for a diagnosis regardless of her self-reported difficulties with faces, e.g., failing to recognize her wife, daughter, or father. This argument has been adapted from McIntosh and Rittmo (2021)

To illustrate this, imagine we test excluded DP cases on a face recognition task, and plot their scores with classical prosopagnosia cases. We may find that their average level of impairment is only – 2 SDs, i.e., excluded cases merely inhabit the top half of the normally distributed ‘prosopagnosia’ group (white Gaussian distribution on the left, Fig. 1). If this is the case, then it means that our face recognition task only has 50% statistical power for detecting an effect (i.e., correctly confirming the presence of developmental prosopagnosia) when using the single-case approach (McIntosh & Rittmo, 2021). Conversely, there will be a 50% chance of committing a type II error (McIntosh & Rittmo, 2021), i.e., a rejection of someone with prosopagnosia as neurotypical, when they actually do suffer from this condition. This could explain why half of all potential prosopagnosia cases fail to meet single-case criteria on tasks such as the CFMT: it is because at the group level, they are only impaired – 2 SDs below the control mean (Fig. 1, dashed vertical line).

To demonstrate this problem further, imagine neurotypical (NT) participant Eleanor reports no trouble with faces, and displays above average abilities on a face processing task, scoring 1 SD above the control mean (Fig. 1 black circle). Now imagine Eleanor lives her life again, but this time we induce in her the genetic or developmental atypicality that results in her developing DP. This causes severe real-world problems for those with DP, but our task is imperfect at capturing this, with the average DP level of impairment only reaching – 2 SDs below the control mean (Fig. 1, dashed vertical line). If this is the case, then DP Eleanor will now report severe difficulties in daily life, has experienced a – 2 SD degradation in her face recognition abilities as assessed on this task, but will never reach below the – 2 SD cut-off required for a diagnosis, i.e., she only scores –1 SD below the control mean (Fig. 1, white circle)^2^. When using the current single-case approach for diagnosing prosopagnosia, Eleanor’s subjective experiences simply do not matter. If she fails to meet diagnostic criteria on the CFMT, she is categorized as not having prosopagnosia, even though this false negative has occurred because of low single-case power.

We believe this exclusion needs to end. So long as Eleanor is not suffering from another condition that could explain her difficulties with faces (e.g., schizophrenia, where individuals can erroneously judge faces as known or not; Bortolon et al., 2015; Guillaume & Thomas, 2021; Pelletier et al., 2005), then we should take her first-hand accounts at face value, and accept they warrant further investigation.

Low power is not just an issue when researchers are using a single test, such as the CFMT, to diagnose prosopagnosia. Many authors require significant impairments at the single-case level on two (e.g., CFMT and FFT, Fisher et al., 2020; Pertzov et al., 2020; Stumps et al., 2020; Wilcockson et al., 2020) and sometimes three (CFMT, CFPT and a prosopagnosia questionnaire, Mishra et al., 2020) measures before they will confirm a diagnosis. We know that roughly 50% of potential DP cases will fail to meet diagnostic criteria when assessed using the CFMT (Bate et al., 2019a; Burns et al., 2014a; Murray & Bate, 2020). If we then add additional conservative single-case requirements on a second or third task, but these measures only average around 50% power for detecting impairment at the individual level too (Bate et al., 2019a), then power for diagnosing DP will rapidly reduce even further. This will result in the exclusion of many potential DP cases who are reporting severe difficulties in daily life, simply because of low statistical power.

### Exclusion means we will never know the prevalence or distribution of DP

**Fig. 2:**
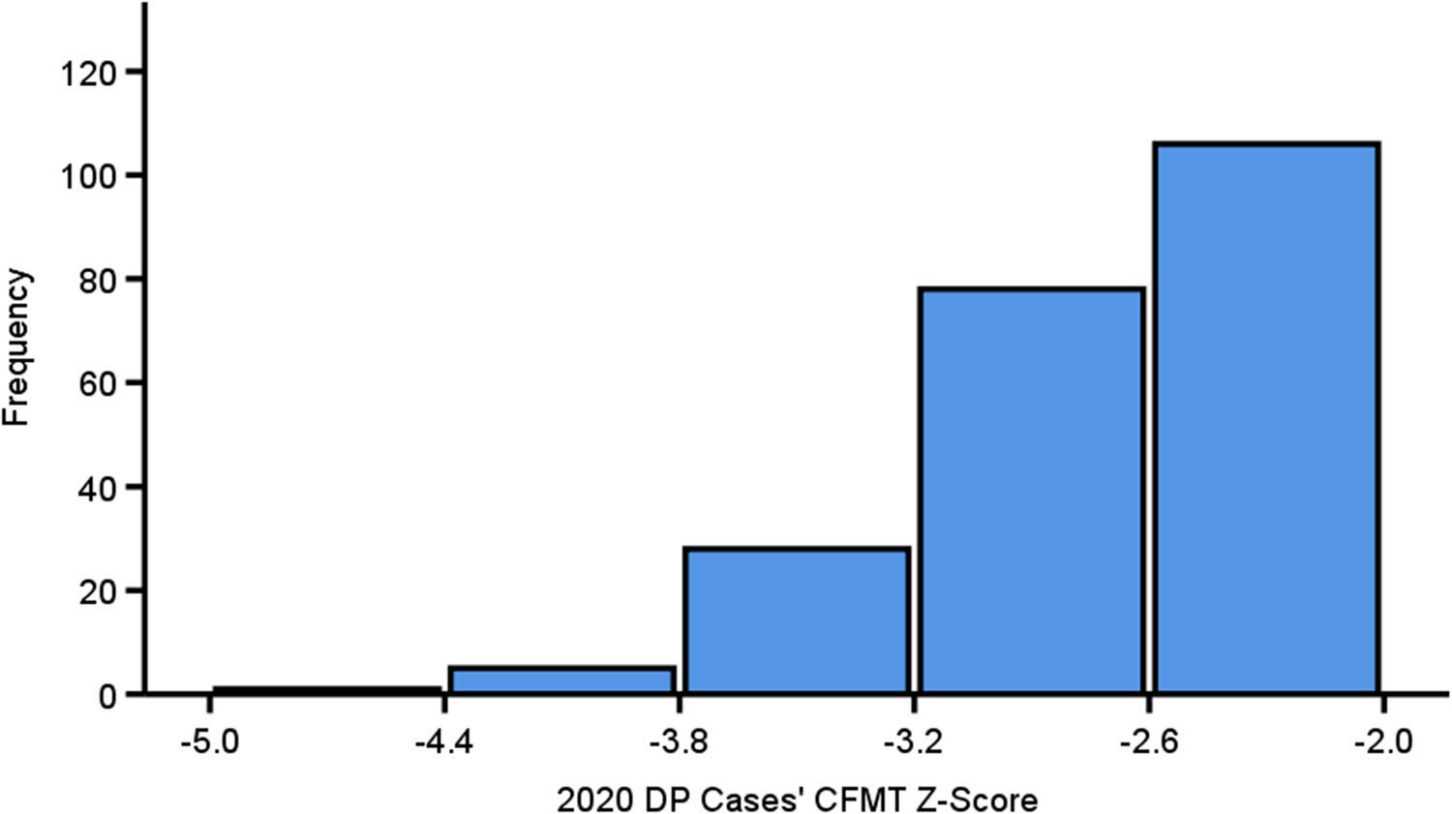
The distribution of every DP case’s *z*-scores that were significantly impaired (i.e., > – 2 SDs below the control mean) on Cambridge Face Memory Test in all papers published in 2020. The distribution was significantly left-skewed [*W*(218) = .93, *p* < .001] with a higher frequency of cases near the – 2 SD cut-off below the neurotypical mean. This suggests that those with DP merely inhabit the quantitative tail-end of the normal distribution (Barton & Corrow, 2016). However, researchers exclude 50% of all people who believe they have DP, so we do not know what the distribution would look like when they are added in. Some studies were not included because they did not report individual CFMT data (Bylemans et al., 2020; Djouab et al., 2020; Gerlach et al., 2020; Tian et al., 2020)

Exclusion of DP cases also poses problems when it comes to assessing its prevalence. There is considerable debate as to whether those with DP reflect the quantitative bottom end of the normal distribution of face recognition abilities in the general population, or are their own discrete population (Arizpe et al., 2019; Barton & Corrow, 2016; Bate & Tree, 2016; Bobak et al., 2017; Tardif et al., 2019)^3^. If the former is true, then the distribution of those with DP should exhibit a left-skew, owing to the fact that the frequency of such cases will increase as the level of impairment becomes milder, with the highest frequency of cases appearing just below the cut-off for a diagnosis at – 2 SDs below the control mean, i.e., if those with DP largely reflect the bottom 2.5% of the normally distributed general population (Barton & Corrow, 2016; Bate & Tree, 2016). To assess this, we plotted the distribution of all DP cases who met the commonly required criteria of a > – 2 SD impairment on the CFMT from the studies in Table 1. Strikingly, we found that these cases do indeed exhibit a significant left-skew distribution [Fig. 2, *W*(218) = .93, *p* < .001], which we would expect if DP merely reflected the tail-end of the neurotypical distribution.

However, these individuals only represent roughly half of all potential prosopagnosia cases (Bate et al., 2019a; Burns et al., 2014a; Murray & Bate, 2020); the other half fail to score below the – 2 SD cut-off required to attain a diagnosis. If we assume these excluded individuals *do* suffer from DP, but the CFMT fails to capture this sufficiently to provide a diagnosis, then these cases will never be included in the distribution. This means that we will not be able to judge if this group are distributed across the next 2.5% portion of the tail-end of the neurotypical distribution, i.e., the bottom 5% of the general population having prosopagnosia. Alternatively, when all DP cases are plotted together, they may reflect a normally distributed group that is distinct from the neurotypical population, with the excluded cases inhabiting the distribution’s top end (i.e., the white Gaussian distribution in Fig. 1). We therefore need to stop excluding cases otherwise we will never know developmental prosopagnosia’s true prevalence and distribution.

### The CFMT test–retest reliabilities mean DP diagnosis status can change on different days

Another issue with researchers relying on the CFMT for a diagnosis is that its test–retest reliabilities are imperfect: some participants’ test scores will fail to meet criteria when tested on one day, but will then meet criteria when tested on another (Murray & Bate, 2020). The statistical evaluation of impaired versus spared using absolute cut-offs, either through the Crawford’s *t* test or two standard deviations below a control mean, are unlikely to be terribly meaningful when the margins are extremely thin. For example, the CFMT is commonly used to diagnose prosopagnosia when a potential case scores 42 trials or fewer (which is > –2 SDs from the control mean) out of 72 correct (Table 1). By contrast, a potential DP case would be classified as spared in their memory abilities if they score 43 trials or above correct. Can we honestly state this is a functionally relevant difference for a diagnosis of prosopagnosia? We do not believe so when the excluded cases are, at least anecdotally, reporting comparable problems in daily life to those who do meet criteria.

### DP literature’s grey population

From a knowledge perspective, we know virtually nothing about excluded DP cases. This is because when they fail to meet commonly used criteria, they are blocked from participating in our research, resulting in their absence in the literature. In meta-analysis research there is a grey literature (Conn et al., 2003; McAuley et al., 2000; Paez, 2017; Rothstein & Hopewell, 2009), i.e., a collection of studies that are never published, which can make it difficult for meta-analysis researchers to accurately understand any given effect they are studying. The excluded DP cases are, in essence, the prosopagnosia field’s very own grey literature, although instead of being studies that are never published, they are a grey population that are almost never reported on.

Owing to this exclusion, we do not know if these cases are experiencing a milder form of the disorder, or if they are exhibiting difficulties in a specific aspect of face recognition, e.g., maybe they can recognize faces over a short retention interval, such as when tested on the CFMT, but not over longer periods of time (e.g., morning to afternoon, day to day, or week to week; McKone et al., 2011). Also, why are they reporting problems in daily life but not on our objective tests? Could another measure beyond the CFMT, FFT and CFPT be more accurate in capturing their daily difficulties with faces, e.g., Prosopagnosia Index (Shah et al., 2015), the Oxford Face Matching Task (Stantic et al., 2022), Glasgow Face Matching Test (Burton et al., 2010; White et al., 2022), Benton Test (Murray et al., 2021; Rossion & Michel, 2018; Wang et al., 2020) or UNSW Faces Test (Dunn et al., 2020)? All these questions are unanswered because excluded cases are rarely reported upon by researchers.

### Exclusion will potentially inflate effect sizes of impairments in DP

Let us assume that excluded prosopagnosia cases do have prosopagnosia and are suffering from a milder^4^ form of the disorder. It is common for tests such as the CFMT to be predictive of other cognitive abilities, e.g., emotion processing, Biotti & Cook, 2016), reading (Burns & Bukach, 2021) and object expertise (Fry et al., 2020). This means that when we exclude the milder DP cases from our research, we will overestimate DP cases’ effect sizes of impairments in other abilities. For example, one study found that emotion recognition difficulties in DP were predicted by more severe face memory problems when assessed using the CFMT (Biotti & Cook, 2016). If we populate the top end of the DP cases’ distribution with excluded cases who perform much better on the CFMT, then we should expect to find estimates of emotion recognition difficulties in DP becoming smaller.

Incorporating excluded cases is therefore important for assessing the broader cognitive difficulties those with DP are experiencing in daily life, as right now, they may not be as impaired in other aspects of cognition as we believe. Also, their inclusion is important for testing models of visual recognition that posit the extent to which different visual functions are integrated with one another, e.g., does face, word, and object recognition rely upon shared cognitive and neural processes, or are they entirely dissociable (Behrmann & Plaut, 2013, 2014; Bukach et al., 2006; Burns et al., 2017b; Dehaene, 2005; Dehaene & Cohen, 2007; Dehaene et al., 2010; Gauthier et al., 1999; Gauthier et al., 2000; Gerlach et al., 2022; Gerlach & Starrfelt, 2022; McKone et al., 2007)?

### Exclusion may underestimate the efficacy of treatments for DP

Exclusion also presents problems with treatments. To date, there are numerous ways in which prosopagnosia cases can potentially improve their face processing abilities (Bate et al., 2014; DeGutis et al. 2014a, b; DeGutis et al., 2011). For example, the hormone oxytocin, commonly referred to as the ‘love’ or attachment hormone due to its social bonding effects (Campbell, 2008; Kosfeld et al., 2005; Nelson & Panksepp, 1998), has been shown to aid social information processing in many neurodevelopmental groups (Keech et al., 2018; Wang et al., 2019b), including developmental prosopagnosia (Bate et al., 2014). Face training paradigms designed to rehabilitate face processing skills, in some cases, may potentially help alleviate their troubles with faces (Bate et al., 2022; DeGutis et al., 2011; DeGutis et al., 2014a, b). Finally, navigation training (i.e., understanding your spatial location in an environment) may even help improve face recognition abilities (Bate et al., 2019b).

Unfortunately, as excluded prosopagnosia cases are never tested with these treatments, we have no idea if they might work for them too. If treatments are more successful for DP cases with milder levels of cognitive impairment, then excluded prosopagnosia cases may be better candidates for rehabilitation due to their residual face processing abilities. Researchers could therefore be underestimating the extent to which prosopagnosia can be helped, simply because we are excluding those who may gain the most from current treatments.

### Heterogeneity of diagnostic methods will hinder replication efforts

The field of psychology has been mired in a replication crisis over the last several years, where previously published effects have proven difficult to replicate (Camerer et al., 2018; Collaboration, 2015). While there are few failed replications we know of in the DP literature (e.g., Behrmann et al., 2005; Duchaine et al., 2007b; Leib et al., 2012; Robson et al., 2018), if researchers are using different cut-offs for determining a diagnosis of DP in their samples, then there is a possibility that failed replications could arise, even when tested effects are real.

For example, Table 1 shows remarkable heterogeneity between how labs diagnose DP. If researchers need impairment on one specific task (e.g., the CFMT: Gerlach et al., 2020), then they will be able to include 50% of potential DP cases in their studies, as on average, 50% of participants are sufficiently impaired at the single-case level to attain a diagnosis via the CFMT. However, if researchers need impairment on two specific tasks (e.g., the CFMT and FFT, Stumps et al., 2020; Wilcockson et al., 2020), or three (e.g., Fry et al., 2020), then these authors will test a much smaller sample of the most severely impaired cases who can meet all these criteria.

If these face processing impairments are related to difficulties in other aspects of cognition, as is often the case (Biotti & Cook, 2016; Biotti et al., 2017b; Burns & Bukach, 2021), then it is inevitable that the studies testing the most severely impaired DP cases will be more likely to detect other cognitive impairments than studies using more liberal diagnostic criteria, at least when sample sizes are equal. Similar issues will be apparent when there is variability in the cut-offs used too. This means that if researchers try to replicate an effect that was previously identified in a group of severely impaired DP cases (e.g., requiring impairment on three measures), but they themselves use DP cases with milder impairments (e.g., just one measure or more liberal SD cut-offs), then their chances of replicating the effect will be diminished.

### Excluding DP cases is upsetting

Finally, another reason to end the exclusion of DP cases in our work is because of the negative impact this has upon these individuals. As mentioned, at least half of those who believe that they have this condition are not eligible for our research because they do not meet the stringent criteria required for a diagnosis. When I (the first author) started testing people with prosopagnosia early in my career and this occurred, I felt terrible, as I had to explain to them that while I personally believed they have prosopagnosia based upon their experiences from our interview, they were not eligible to participate in our research. You could see the disappointment on their faces; it was obvious that they felt like their difficulties were not being validated, that they were being rejected. They would ask questions like “If I’m not eligible for research, then what’s wrong with me?”, “Why can’t I recognize my family members?”. I could not at the time, 10 years ago now, provide any answers because researchers knew nothing about them. Fast forward to the present day and we still do not have any answers to give them as we continue to exclude them from our work.

We should add that these experiences have not only happened with the cases we have tested. Many people who believe that they have prosopagnosia have contacted me over the years and reported having had the same experiences with other prosopagnosia labs: they did not meet the criteria for a diagnosis so would not be used for their research. This has often left them feeling terribly upset, angry and frustrated. Similarly, many prosopagnosia researchers I have spoken with have had the same experiences with excluded cases. The failure to acknowledge these individuals as having prosopagnosia has, in my experience of speaking with them, been potentially damaging for how they view our field of research.

Of course, we should stress we do not think a participant’s frustration at not receiving a diagnosis should alone be reason enough to include them in our work. Maybe they do suffer from a yet unidentified issue that is distinct from prosopagnosia. However, because we never conduct research on these individuals, we can never hope to understand what these problems are.

### What are the characteristics of excluded DP? The present study

In summary, there are many problems associated with DP exclusion, and they will afflict virtually every aspect of prosopagnosia research if it continues. We therefore hope readers can see the necessity for ending this practice. To support this proposal, we wanted to formally assess whether excluded individuals do, as a group, exhibit deficits when tested through commonly used diagnostic tasks, i.e., the CFMT, CFPT, and FFT. If they do, then this would show that these cases’ objective problems with faces warrant further investigation.

Here we used the CFMT as our diagnostic tool to categorize prosopagnosia participants as *Classical* DP cases (i.e., those who are impaired at the CFMT > – 2SD below the control mean), or *Excluded* cases (i.e., those who score better than – 2SD below the control mean), as it is the most widely used test and cut-off for diagnosing DP (Table 1). This approach may result in a slightly more liberal inclusion of some cases as classical DP than if we required impairment on a second test (e.g., the FFT). However, given potential differences in how the FFT is used across labs, we thought using the CFMT alone to diagnose prosopagnosia here would be simpler and allow our findings to be more easily replicated. We do though ourselves replicate some of our own results by using a previously published group of DP cases from Bate et al. (2019a), categorizing them as classical DP (i.e., impaired on CFMT *and* FFT) or excluded (i.e., those that do not meet this criteria) to ensure the conclusions are largely the same even with commonly used conservative criteria of impairment on two tasks.

Anecdotally speaking, those who fail to meet criteria for a diagnosis also report symptoms that are comparable to those who do meet the criteria, e.g., failing to identify people they should, mixing up celebrities. We therefore wanted to assess these difficulties quantitatively, i.e., do excluded cases report similar problems in daily life to those that do meet criteria for a diagnosis, or are their symptoms milder, reflecting their weaker levels of impairment on the CFMT. To measure this, we used the prosopagnosia index questionnaire (PI20; Shah et al., 2015), which asks participants to report how strongly they agree that they experience prosopagnosia symptoms in their lives. Moreover, we wanted to test whether the PI20 was more effective for detecting potential DP cases at the single-case level, when compared to other tasks such as the FFT, CFMT, and CFPT.

Finally, we wanted to assess whether excluded cases exhibit problems in holistic perception. It is thought that effective face recognition is attained through the perception of a face as a single, unitary whole item (i.e., holistic), where the features’ configural relationships interact with one another, rather than being viewed as distinct, disparate parts that do not (McKone & Yovel, 2009; Richler et al., 2011; Richler et al., 2012). Holistic perception is commonly reported as being the source of prosopagnosia cases’ difficulties with faces (Avidan et al., 2011; DeGutis et al., 2012; although see Biotti et al., 2017), so it will be interesting to gauge if excluded prosopagnosia cases are suffering milder atypicalities with this than classical cases.

## Methods

### Participants

All prosopagnosia participants contacted us after seeing requests for participants on social media, email campaigns, plus adverts and word of mouth within the university we work. This resulted in us recruiting a total of 62 DPs, including nine males and three people who neither identified as male nor female. Ages ranged between 18–72 years (*M* = 41.5; *SD* = 14), with all cases reporting lifelong difficulties with faces and no obvious historical reason for it being acquired. Due to COVID-19 restrictions banning in-person testing, all tasks were completed online via the Testable experiment builder. One of the 62 DP cases failed two attention check trials during our neuropsychological assessments, and so was excluded from all analyses. Another DP case failed one attention check, but we still included them because it seemed reasonable to assume they may have just made a mistake on that trial, particularly as they were accurate on the other check. A final DP case did not seem to move the faces during the CFPT, but they did perform within the DP range on the CFMT and FFT, and passed both attention check questions, so we surmised they may have just had difficulties with their mouse (62-year-old female who made 96 errors on upright and inverted CFPT, Table 2). We therefore excluded her from any CFPT-related analyses. This left us with 61 DP cases (Table 2) for all tests but the CFPT-related measures (*DP n* = 60).

**Table 2:** Neuropsychological test results of the 61 DP cases. Columns indicate: Prosopagnosia index questionnaire (PI20), Famous Faces Test (FFT), Cambridge Face Memory Test (CFMT), Cambridge Face Perception Test upright scores (CFPTup), Cambridge Face Perception Test inverted scores (CFPTinv) and the Cambridge Face Perception Test Holistic Perception measure (Holistic Perception)

DP Diagnosis	Age	Sex	CFMT	CFPTup	CFPTinv	Holistic Perception	FFT	PI20
Excluded	54	Female	67	32	80	1.50	21	**78**
Excluded	31	Female	63	56	70	.25	18	**65**
Excluded	51	Female	62	26	62	1.38	14	**88**
Excluded	33	Female	58	42	68	.62	20	**77**
Excluded	24	Female	58	30	78	1.60	12	62
Excluded	52	Male	57	44	62	.41	22	**84**
Excluded	40	Female	57	22	56	1.55	23	**66**
Excluded	20	Female	57	38	46	.21	18	**91**
Excluded	52	Female	56	20	56	1.80	26	**82**
Excluded	22	Female	54	50	**90**	.80	**5**	**83**
Excluded	51	Female	54	46	76	.65	15	**85**
Excluded	52	Female	54	42	48	.14	12	**79**
Excluded	35	Female	51	60	76	.27	18	**89**
Excluded	31	Male	50	52	66	.27	17	**73**
Excluded	66	Male	50	40	60	.50	12	**75**
Excluded	31	Male	49	24	70	1.92	10	**78**
Excluded	67	Female	49	46	56	.22	9	**76**
Excluded	24	Female	48	56	76	.36	**4**	**84**
Excluded	54	Female	48	**78**	**84**	.08	**2**	**95**
Excluded	44	Female	48	**64**	50	– .22	9	**86**
Excluded	33	Female	47	**86**	74	– .14	14	**93**
Excluded	28	Other	46	**96**	**88**	– .08	11	**86**
Excluded	32	Female	46	44	50	.14	20	**90**
Excluded	34	Female	46	30	52	.73	22	**89**
Excluded	40	Female	46	38	42	.11	19	**76**
Excluded	40	Female	46	52	72	.38	12	**84**
Excluded	72	Female	46	48	64	.33	**8**	**89**
Excluded	62	Female	45	**96**	**96**	.00	**8**	**82**
Excluded	56	Female	45	54	46	– .15	**6**	**82**
Excluded	39	Male	44	**64**	82	.28	15	**78**
Excluded	41	Female	44	34	54	.59	13	**85**
Excluded	39	Male	44	28	84	2.00	10	**86**
Excluded	50	Female	43	52	82	.58	12	**91**
Excluded	48	Female	43	54	54	.00	**8**	**80**
Classical	52	Female	**42**	54	70	.30	22	**73**
Classical	18	Female	**42**	40	42	.05	11	**76**
Classical	71	Female	**42**	44	66	.50	11	**67**
Classical	22	Other	**41**	48	80	.67	20	**83**
Classical	43	Male	**41**	58	70	.21	11	**87**
Classical	66	Female	**41**	48	68	.42	**6**	**73**
Classical	31	Male	**40**	52	62	.19	**5**	**87**
Classical	43	Male	**39**	32	60	.88	17	**71**
Classical	38	Other	**38**	38	66	.74	**7**	**79**
Classical	35	Female	**38**	38	82	1.16	9	**85**
Classical	44	Female	**38**	56	54	– .04	14	**68**
Classical	22	Female	**37**	**78**	**88**	.13	**6**	**83**
Classical	30	Female	**37**	**70**	**86**	.23	13	**81**
Classical	32	Female	**36**	52	80	.54	11	**94**
Classical	64	Female	**35**	46	52	.13	**6**	**87**
Classical	32	Female	**35**	42	82	.95	**2**	**80**
Classical	27	Female	**34**	**76**	50	– .34	11	**94**
Classical	49	Female	**34**	**78**	68	– .13	**6**	**79**
Classical	34	Female	**33**	54	**96**	.78	23	**80**
Classical	48	Female	**33**	**62**	84	.35	14	**94**
Classical	19	Female	**32**	48	76	.58	11	**82**
Classical	20	Female	**31**	54	**92**	.70	14	**75**
Classical	44	Female	**30**	60	50	– .17	10	**82**
Classical	42	Female	**29**	**74**	**94**	.27	**5**	**89**
Classical	44	Female	**29**	**76**	**76**	.00	14	**81**
Classical	49	Female	**22**	**78**	**104**	.33	13	**85**
Classical	62	Female	**21**	**86**	**88**	.02	**4**	**92**

Bold ﻿indicates impairment of two SDs using norms in the literature (CFMT: Duchaine & Nakayama, 2006; all CFPT scores: Duchaine, Germine, & Nakayama, 2007a), from our control sample (FFT, Holistic Perception), or from recommended cut-offs (PI20: Shah et al. 2015)

Fifty-two control participants were recruited from the online experiment builder Testable’s subject pool. Their ages ranged from 22–68 years (*M* = 38.8; *SD* = 11.3), including 31 males. While there were many more males in the control group, men are typically poorer at recognizing faces than women (Herlitz & Lovén, 2013; Mishra et al., 2019). If this gender difference is also true in prosopagnosia, then the gender disparity means that we are likely to slightly underestimate the levels of impairments in our prosopagnosia cases here. We realize readers may be concerned this could affect our conclusions, so we performed between gender *t* tests on every face processing task measure across control participants. This was to ensure there were no gender differences in our control sample that may unduly influence any neurotypical vs. DP group comparisons. Importantly, these analyses revealed no evidence for gender-related effects [all *ps* > .08; *BF*_*10*_ ≤ 1]. We therefore do not take gender into account in any of our Results section analyses on our own data.

Three control males disclosed difficulties with faces in daily life, where they reported regularly failing to recognize people they personally know and celebrities, which are the primary characteristics of prosopagnosia. These issues were reflected in their prosopagnosia symptomology (i.e., the prosopagnosia index questionnaire scores) appearing close to, or within, the prosopagnosia range (PI20 scores: 74, 68, and 63; cut-off for DP is typically > 64), and so were excluded from all analyses. It is worth noting that this would put the prevalence of prosopagnosia at 5–6%, which is closer to the higher end estimate of 5% reported by Bennetts et al. (2017) than the commonly reported 2–2.5% (Kennerknecht et al., 2006; Kennerknecht et al., 2008).

A female control participant was excluded because she did not move any faces during the Cambridge Face Perception Test, gave largely minimal answers on the famous faces test (e.g., ‘woman’, ‘man’), and scored below chance levels on the Cambridge Face Memory Test (i.e., 21 correct out of 72 trials, with chance at 24 correct). We should stress, including the participants we removed from our dataset had no impact upon the pattern of effects we observed with respect to excluded cases exhibiting group-level impairments in comparison to controls. Also, a one-way ANOVA revealed no significant differences between the ages of the remaining excluded prosopagnosia group, the classical DP group and control participants [*F*(2, 106) = .86, *p* = .43] with Bayesian analyses supporting the null hypothesis [*BF*_*10*_= .15].

DP cases who score more than 2 SDs below the control mean on the CFMT have a mean level of impairment of – 2.7 SDs (*SD* = .53; taken from data in Fig. 2). If half of DP cases fail to score more poorly than 2 SDs below the control mean, then it is possible the mean level of DP impairment on this test, when excluded cases are included, could be – 2 SDs. We therefore thought it reasonable to assume the excluded cases would have an average level of impairment of 1.3 SDs below the control mean (i.e., the same distance from – 2 SDs as what the cases who do meet criteria exhibit). The smallest *Cohen’s f* for such between group analyses (i.e., classic DP vs. excluded DP vs. controls) would be .64. Using this *Cohen’s f* in a power analysis in G*Power suggested for a one-way ANOVA, we would only need 13 participants in each group to obtain over 80% power with alpha set to .05. We therefore aimed for 20 classic and 20 excluded DP cases, and planned to roughly match the controls afterwards. We managed to recruit well beyond this number though (*Excluded DP n* = 34, *Classical DP n* = 27, *Control n* = 48). A power analysis suggested that with 109 participants we would be able to detect medium to large effects sizes (i.e., *Cohen’s f* > .3) with 80% power and alpha set at .05. Deidentified data for controls and DP cases, plus our Famous Faces Test, is available on the Open Science Framework (https://osf.io/tx5av/?view_only=a69e476ec5614833a4075eaff01e0d4e). When any non-significant differences occurred between the groups in our Frequentist analyses, we ran additional Bayesian analyses to assess the level of evidence in favour of the null hypothesis. None of our experiments or hypotheses were pre-registered.

### Procedure and materials

All participants completed a battery of tests that are commonly used diagnose developmental prosopagnosia. These included the Cambridge Face Memory Test (CFMT; Duchaine & Nakayama, 2006), the Cambridge Face Perception Test (CFPT; Duchaine et al., 2007a), a Famous Faces Test (FFT) and the Prosopagnosia Index questionnaire (Shah et al., 2015). All DP cases’ neuropsychological data are presented in Table 2.

The Cambridge Face Memory Test (CFMT; Duchaine & Nakayama, 2006) is the most widely used test for diagnosing developmental prosopagnosia (Table 1). It requires participants to identify one of six previously studied target faces from a line up containing the target and two lures. The final section of the CFMT asks participants to identify the faces with noise present, thereby enhancing the difficulty. There are 72 trials, with one point given for each correct trail. This test is also commonly used to assess face memory abilities in neurotypical samples (e.g., Bate et al., 2019c; Dennett et al., 2012; McKone et al., 2012) in addition to diagnosing DP (Bate et al., 2014; Bate & Tree, 2016; Biotti & Cook, 2016; Biotti et al., 2017b). Based on norms from prior work (Duchaine & Nakayama, 2006), we categorized 44% of our prosopagnosia cases as classical prosopagnosia (i.e., scoring beyond more than – 2 SDs below the control mean) and 56% as excluded prosopagnosia (i.e., those who failed to meet this criterion for a diagnosis).

The Cambridge Face Perception Test (Duchaine et al., 2007a) requires participants to arrange six morph continua faces in order of similarity to a target face. Scores are computed as errors from where the face should have been placed with respect to its similarity to the target. There is an upright and inverted portion of this task, with eight trials in each of these conditions. We used norms from Duchaine et al. (2007a) to identify 23% DP cases were impaired on this task (Table 2).

We created a measure of holistic perception using CFPT upright and inverted scores by performing the following calculation: (CFPTinverted - CFPTupright)/CFPTupright; Russell et al., 2009). This is because upright face processing is thought to be heavily reliant upon viewing the face as a single whole entity through our everyday experiences with faces (i.e., they are almost always encountered upright), whereas inversion is thought to disrupt these ‘typical’ face processing abilities, requiring participants to engage in feature-based processing to detect similarities (i.e., relying upon matching between a nose, or mouth corner, Valentine, 1988). By removing the upright errors from the inverted, it is thought that the remaining value indexes holistic perception (Russell et al., 2009), with larger positive values indicative of superior abilities.

Participants also completed a famous faces test (FFT). This comprised 30 trials, where a single image of a celebrity’s face was shown on each trial to the participant. Participants were then required to identify the celebrity with a name or some piece of semantic information to demonstrate that they truly recognized the celebrity, e.g., if the famous actor Tom Cruise’s face was presented and the participant could not remember his name, but could report sufficient semantic information to identify him, such as “famous actor in Mission Impossible”, then the trial would be scored as correct. Unfortunately, due to a programming oversight, we did not ask participants if they were familiar with the celebrities afterwards, so were unable to confirm if the reason why participants failed to recognize any celebrity was because of an impairment in face recognition, or because they simply were not familiar with them. We suspect this was why a relatively small proportion of DP cases met the single-case level impairment (26%) in comparison to a recent paper (75%; Bate et al., 2019a). We therefore thought it reasonable to replicate our findings using a more conservative diagnostic method (i.e., classic cases scoring more than – 2 SDs below the control means on the CFMT *and* FFT), that is, using data that controlled for famous faces across participants’ ages and familiarity with the celebrities (Bate et al., 2019a). These analyses replicated our pattern of findings here, i.e., excluded cases were always impaired at the group level (see end of Results). Please note, only 15% of DP cases would have been diagnosed if we had demanded ultra-conservative, single case impairment criteria on the CFMT *and *FFT as many labs require. 

The Prosopagnosia Index (PI20, Shah et al., 2015) is a self-reported 20-item questionnaire that assesses the levels of prosopagnosia symptoms in any given individual. Participants endorse, using a five-point Likert scale (i.e., ‘Strongly Disagree’ to ‘Strongly Agree’), statements that quantify the extent to which they suffer from the difficulties those with prosopagnosia experience in daily life, e.g., “I have always had a bad memory for faces”, “I often mistake people I have met before for strangers”, and “I sometimes find movies hard to follow because of difficulties recognizing characters”. Each answer is scored 1–5 (total range 20–100), with higher scores indicative of stronger agreement that the participant suffers from prosopagnosia. The original authors proposed a cut-off of > 64 as being indicative of prosopagnosia (Shah et al., 2015). The PI20 has been validated against two of the most common cognitive tasks used to diagnose prosopagnosia (Shah et al., 2015), i.e., the Cambridge Face Memory Test and the Famous Faces Test.

We tested split-half reliabilities on all measures by correlating the sum of the even trials with the sum of the odd trials. This revealed significant associations for every task and questionnaire: PI20 [*r* = .95, *p* < .001], CFMT [*r* = .88, *p* < .001], FFT [*r* = .87, *p* < .001] and CFPT upright [*r* = .57, *p* < .001].

Ethical approval was granted by Edge Hill University Ethics Review Board, with all work carried out in accordance with the 1964 Helsinki Declaration on human testing. All participants gave their informed consent, including for the authors to publish anonymized data derived from their participation.

## Results

### Excluded prosopagnosia cases report comparable symptoms as classical prosopagnosia

**Fig. 3:**
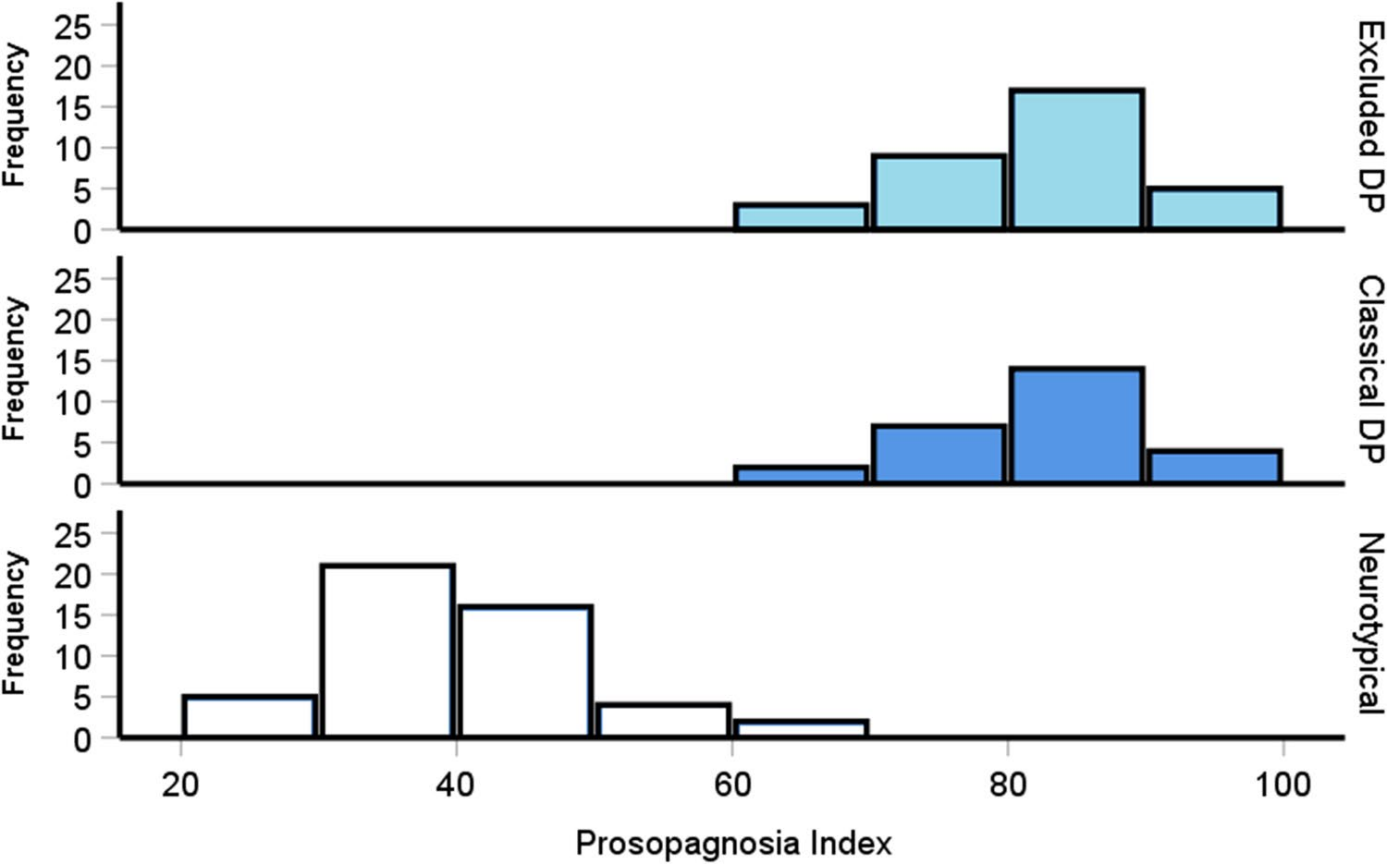
Prosopagnosia index (PI20) distributions for controls plus the excluded and traditional prosopagnosia cases. Scores of 65 or above typically indicates the presence of prosopagnosia. The two DP groups exhibit a similar, overlapping normal distribution. The severity of their reported difficulties with faces was near identical [excluded: *M* = 82, *SD* = 7.8; classical: *M* = 81.7, *SD* = 7.6, *p* = 1], with both impaired relative to controls [*M* = 39.08, *SD* = 9, both *p*s < .001]

In our experience, prosopagnosia cases who fail to meet diagnostic criteria report similar issues with faces at interview as those who do meet criteria. We therefore asked our participants to complete the PI20 questionnaire to quantitatively assess these difficulties in daily life. Figure 3 reveals the prosopagnosia symptoms across both the excluded and classical DP groups appear normally distributed, near identical to one another, and largely distinct from the control group. Shapiro–Wilk tests confirmed that all three groups’ distributions did not significantly deviate from normality [Fig. 3, all *p*s > .11]. We plot histograms throughout so readers can get a sense of the distribution of scores and how they compare across groups.

A one-way ANOVA was performed to compare the severity of the prosopagnosia symptoms between groups, revealing significant differences [*F*(2, 106) = 358, *p* < .001, η^2^ = .87]: both the excluded [*M* = 82, *SD* = 7.8] and classical [*M* = 81.7, *SD* = 7.6] DP groups reported more difficulties with faces than the controls [*M* = 39.08, *SD* = 9, both *p*s < .001], with no differences between the two prosopagnosia groups [*p* = 1]. A Bayesian *t* test indicated substantial evidence favoring a lack of differences between the two prosopagnosia groups’ symptoms [*BF*_*10*_ = .26]. This confirms excluded prosopagnosia cases report near identical difficulties with faces to their classical counterparts.

### Excluded prosopagnosia cases are impaired in face memory

**Fig. 4:**
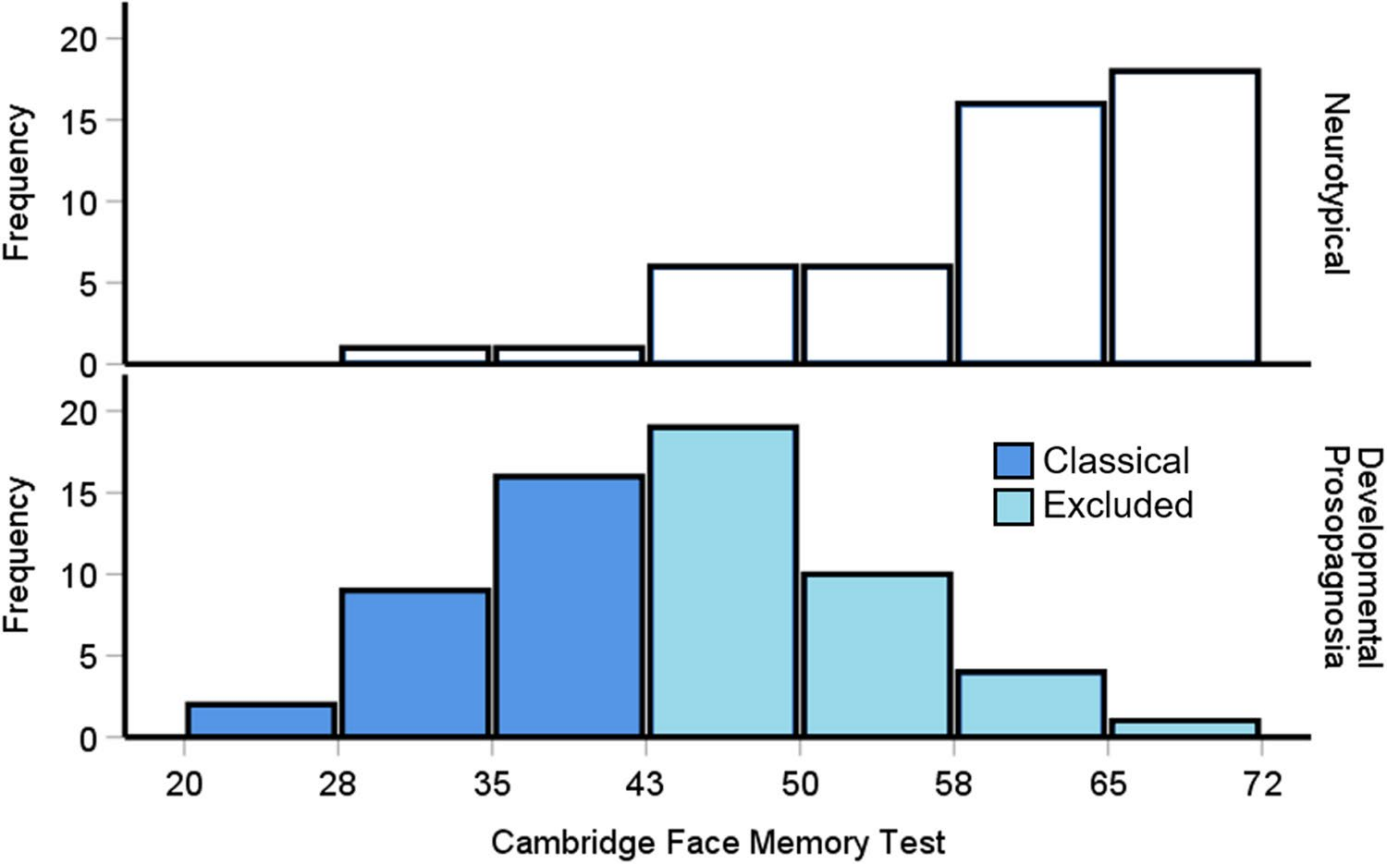
Cambridge Face Memory Test (CFMT) scores for controls (*top*) and the excluded (*light blue, bottom*) and traditional prosopagnosia cases (*dark blue, bottom*). Scores of 42 or below are commonly used to diagnose prosopagnosia. Combining the excluded and classical DP cases appears to complete a normally distributed group. Please note the *top bars* include participants who scored 72 trials correct

The PI20 demonstrated that excluded prosopagnosia cases report problems with faces that are comparable to classical cases. Figure 4 reveals that when the excluded and classical DP cases are plotted together, they comprise a normal distribution, as if they are part of the same group. A Shapiro–Wilk test confirmed the excluded DP cases [*p* = .005], and classical DPs [*p* = .032] exhibited significant right and left skews, respectively. Moreover, the controls displayed a significant left-skew [Fig. 4, *p* = .001], i.e., many neurotypical individuals trended towards high performance on the CFMT.

An issue in the DP field is that excluded cases do not exhibit severe impairments on the CFMT when assessed using a single-case approach (Bate et al., 2019a; Burns et al., 2014a). We therefore wondered if excluded DP cases *as a group* exhibited problems in face memory when measured using the CFMT. Despite the skew, modelling work has shown one-way ANOVAs are robust even when the datasets are not normally distributed (Blanca Mena et al., 2017). We therefore ran a one-way ANOVA to reveal a significant difference between the groups [*F*(2, 106) = 82, *p* < .001, η^2^ = .61]. As expected, the excluded DP participants were significantly impaired [*M* = 50.6, *SD* = 6.33, *p* < .001] relative to the control group [*M* = 59.7, *SD* = 9.8]. This confirms that despite the single-case approach failing to identify abnormalities in excluded DP cases, impairments do exist at the group level. Unsurprisingly, the classical DP cases [*M* = 35.2, *SD* = 5.64] were also impaired relative to the controls [*p* < .001] and excluded DP group [*p* < .001].

As the two DP groups appear to be one, normally distributed group, we wanted to assess what the average level of face memory impairment is when these two prosopagnosia groups were combined. This would yield a much more accurate effect size of impairment for future researchers to base their power analyses upon. A *t* test revealed that when excluded prosopagnosia participants were included, the reduction in performance relative to our controls was roughly – 1.62 SDs [*M* = 43.79, *SD* = 9.78, *t*(107) = 8.41, *p* < .001, Cohen’s *d* = 1.62]. This estimate did extend slightly though to – 1.79 SDs when we used Duchaine and Nakayama’s (2006) norms.

### Excluded prosopagnosia cases are impaired in upright face perception

The Cambridge Face Perception Test is one of the most widely used neuropsychological tests of face processing ability (Duchaine, Germine & Nakayama, 2007a). While it is rarely used to diagnose prosopagnosia (two in 15 papers in 2020), it is commonly employed to categorize prosopagnosia cases as associative (i.e., problems with memory, not perception) or apperceptive (i.e., problems with perception *and* memory). Researchers typically only examine upright trials on this test as they are thought to reflect experience-based face-related processes in the brain (i.e., we usually view faces upright in daily life).

**Fig. 5:**
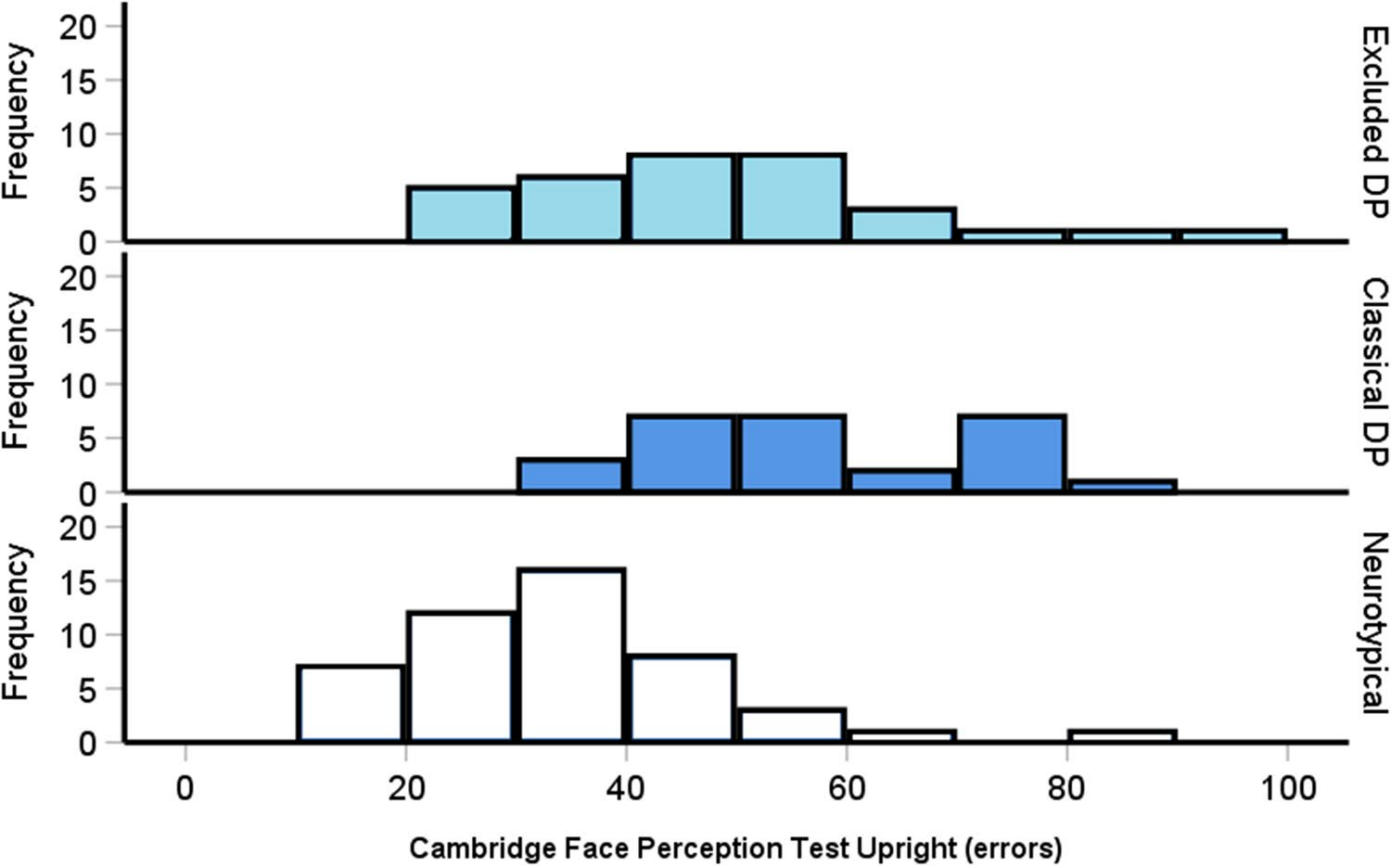
Cambridge Face Perception Test upright errors for controls (*top*) and the excluded and traditional prosopagnosia cases (*bottom*). Errors > 62 are indicative of impairment at the single-case level (> 2 SDs). Both prosopagnosia groups were significantly impaired relative to the controls [*p*s < .001] with the excluded cases performing better than the classical cases [*p* = .038]. The DP cases’ mean errors were – 1.28 SDs poorer than the controls

As with face memory, we wanted to assess whether excluded prosopagnosia cases as a group were associated with deficits on this task. Figure 5 hints that both the excluded and classic prosopagnosia cases are performing substantially poorer on the Cambridge Face Perception Test when compared to the controls. Confirming this, a one-way ANOVA revealed significant differences between the groups [*F*(2, 105) = 21.56, *p* < .001, *η*^*2*^ = .29]: the excluded [*M* = 46.9 errors, *SD* = 17.6, *p* < .001] and classic [*M* = 57.1 errors, *SD* = 15, *p* < .001] DP participants made more face perception errors relative to the controls [*M* = 33.3 errors, *SD* = 14.16]. Moreover, the classical prosopagnosia cases exhibited significantly more errors than the excluded prosopagnosia group [*p* = .038]. Overall, excluded DP cases exhibit considerable problems when attempting to perceive subtle differences between upright faces.

As with the CFMT, we wanted to assess the level of impairment when the two DP groups were combined; the global DP group mean errors were – 1.28 SDs [*M* = 51.5 errors, *t*(106) = 5.91, *p* < .001, Cohen’s *d* = 1.14] poorer than the controls.

For completeness, we ran an ANOVA on the inverted trial data; it is thought that inverting faces disrupts typical face processing routes in the brain that utilize holistic perception, forcing participants to rely instead upon featural processing (Valentine, 1988). A one-way ANOVA revealed no significant differences between any of the three groups [*F*(2, 105) = 2.39, *p* = .097, η^2^ = .04], albeit Bayesian analyses suggested this evidence for the null hypothesis was weak [*BF*_*10*_= .6].

### Excluded prosopagnosia cases’ holistic perception impairments are comparable to classic developmental prosopagnosia cases

**Fig. 6:**
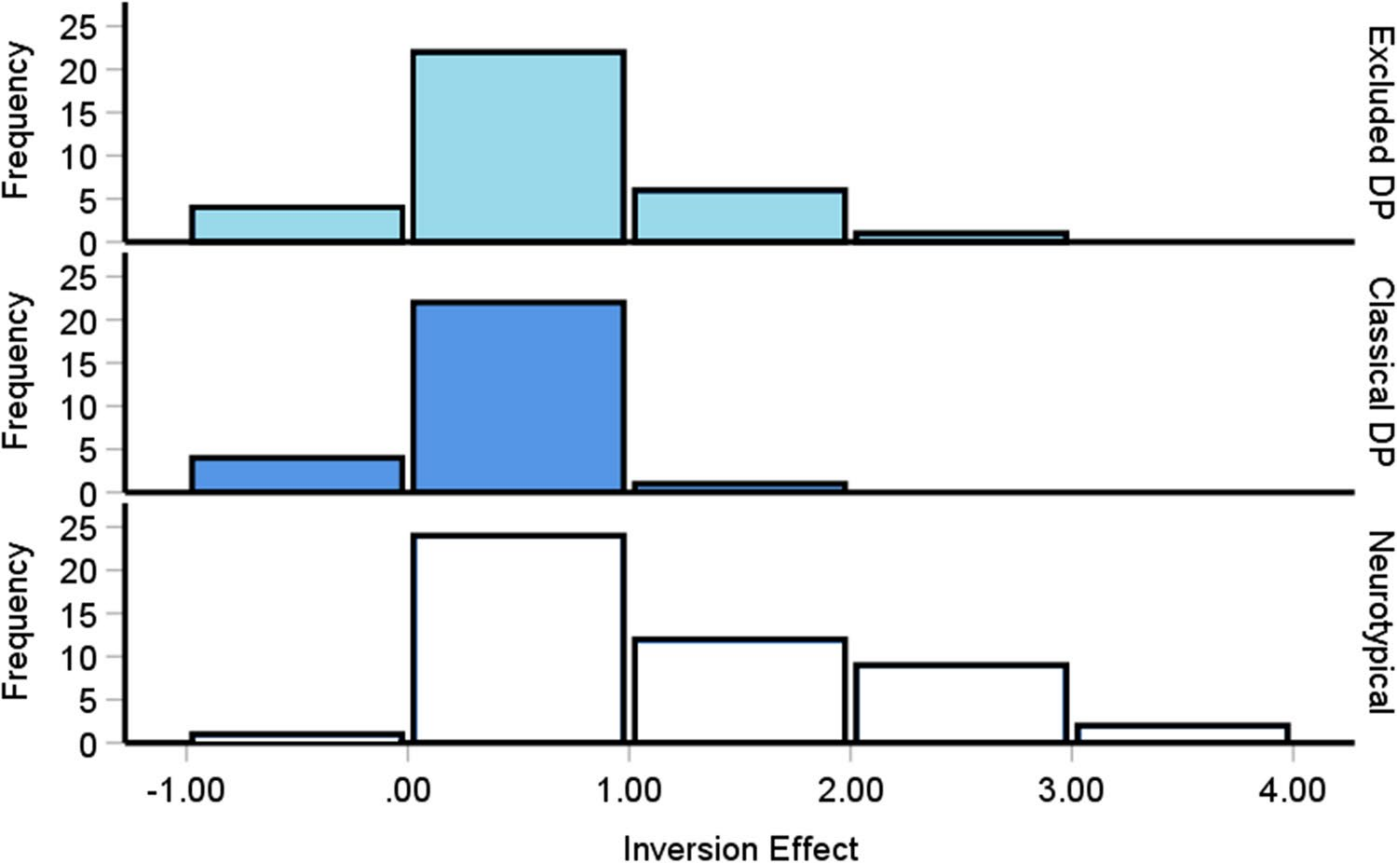
The inversion effect for the neurotypical controls (*bottom*) and the two prosopagnosia groups (*top*). More positive inversion effects is reflective of increasingly superior performance for upright versus inverted faces. Both prosopagnosia groups exhibited significantly lower inversion effects than the controls [Classical DP *p* < .001; Excluded DP *p* = .002], but were not significantly different from one another [*p* = .68]. The DP cases’ mean errors were .76 SDs below the controls

Impairments in the ability to perceive a face holistically, where the features interact to create a unitary percept, has been considered a potential cause of prosopagnosia’s face processing difficulties (Avidan et al., 2011; DeGutis et al., 2012; although see Biotti et al., 2017). We were therefore curious if excluded prosopagnosia cases exhibited similar difficulties with this process as the classical DP cases do, relative to controls. Using our corrected inversion scores from the CFPT as an index of holistic perception, we confirmed this hypothesis through a one-way ANOVA [*F*(2, 105) = 12.6, *p* < .001, η^2^ = .19]: excluded [*M* = .58, *SD* = .64, *p* = .002] and classical [*M* = .35, *SD* = .37, *p* < .001] cases exhibited much smaller inversion effects in comparison to our controls [Fig. 6, *M* = 1.16, *SD* = .9]. There were no differences though between the two DP groups [*p* = .68], although Bayesian analyses suggested this evidence was weak [*BF*_*10*_= .8]. This indicates that as a group, those with excluded prosopagnosia exhibit objective deficits in holistic perception that may be comparable to the individuals with classical DP.

Pooling the two prosopagnosia groups together revealed their mean scores [*M* = .48, *SD* = .54] were .76 SDs below the control mean [*t*(106) = 4.85, *p <* .001, Cohen’s *d* = .94]. In summary, like their self-reported problems with faces, excluded prosopagnosia cases exhibited comparable impairments in holistic perception as those with classic prosopagnosia.

### Excluded prosopagnosia cases exhibit impairments when judging famous faces

**Fig. 7:**
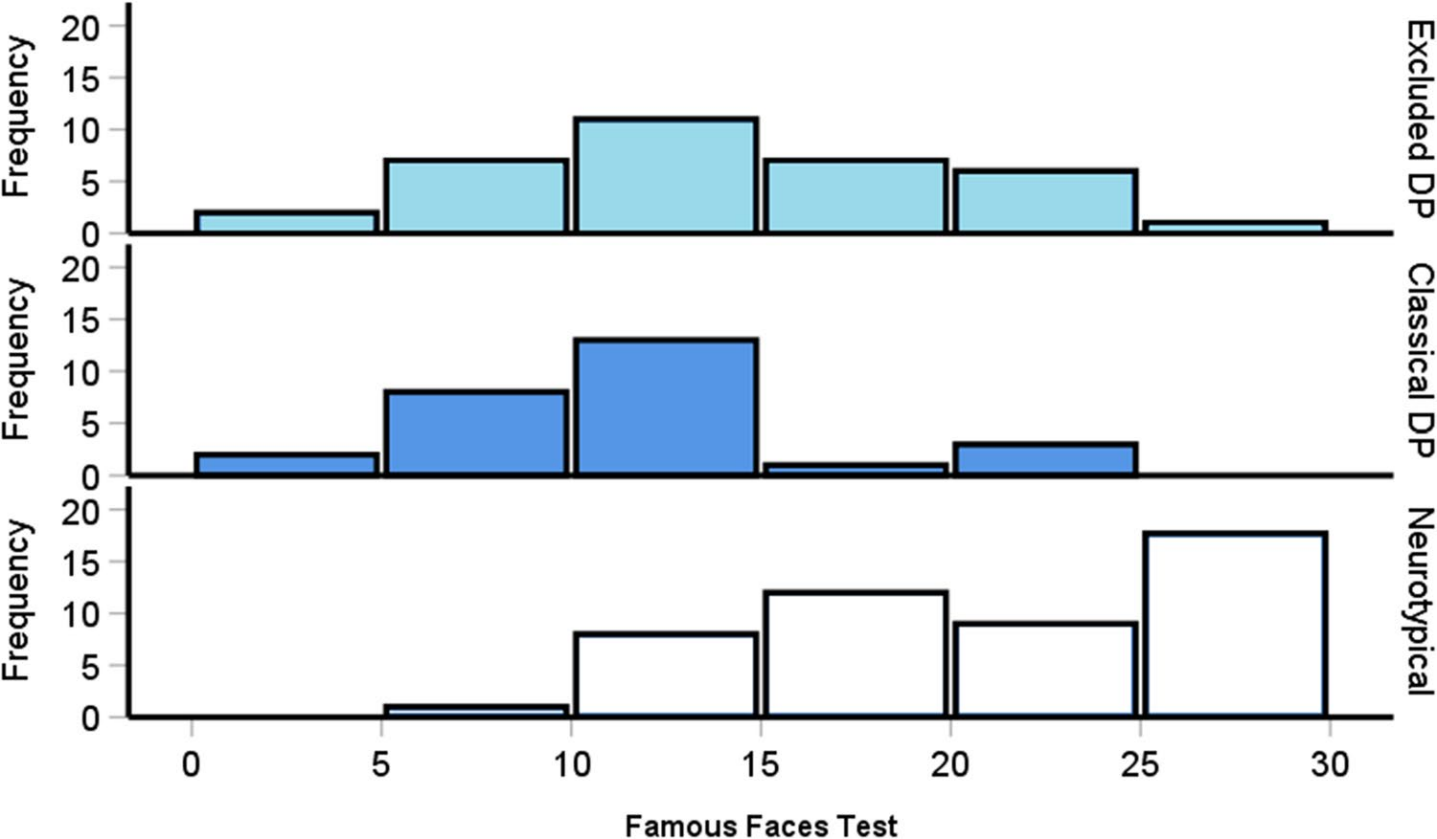
Famous faces test correct trials scores for the neurotypical controls (*bottom*) and the two prosopagnosia groups (*top*). Both prosopagnosia groups exhibited significantly poorer recollection in comparison to the controls [both *p*s < .001] but were not significantly different from one another [*p* = .24]. Please note, the *top bars* include participants who scored 30 trials correct

Excluded prosopagnosia cases exhibited deficits in face perception and recognition, although the latter was for faces that were unknown to the participants. It has been suggested that highly familiar faces, such as friends and celebrities, are to some extent processed in a distinct way from unfamiliar faces (Ellis et al., 1979; Johnston & Edmonds, 2009; Megreya & Burton, 2006). We therefore wanted to confirm that difficulties were also apparent in our excluded cases for known well-known faces. A one-way ANOVA confirmed this [*F*(2, 106) = 27, *p* < .001, η^2^ = .34]: the excluded [*M* = 13.7 correct, *SD* = 5.9, *p* < .001] and classic [*M* = 11 correct, *SD* = 5.4, *p* < .001, Fig. 7] DP participants correctly identified fewer celebrity faces in comparison to the controls [*M* = 20.65 correct, *SD* = 6.2]. Despite the excluded cases identifying roughly two more celebrity faces than our classical cases, this difference was not significant [*p* = .24].

We should add that we did not ask participants to report which names they knew afterwards (i.e., to ensure their misses were because of recognition failure, rather than lack of knowledge). Despite this, we did find significant relationships between our FFT scores and other face-related measures [PI20, *r* = – .6, *p* < .001, 95% CI [– .46, – .71]; CFMT, , *r* = .62, *p* < .001, 95% CI [.49, .72]; CFPT upright, *r* = – .54, *p* < .001, 95% CI [– .39, – .66]; CFPT Inversion Effect, *r* = .46, *p* < .001, 95% CI [.3, .6]]. This suggests that despite not assessing participants’ familiarity with the celebrities, our famous faces test was likely to be tapping into common mechanisms that are impaired in DP.

Despite the relationships with our other tests, one may wonder if we would find the same impairments if we had controlled for familiarity with faces, or differences between the excluded and classical DP groups. One recent study did control for familiarity in their FFT (Bate et al., 2019a), so we categorized their participants as excluded, classical and neurotypical controls in the same way that we did with our sample and ran a one-way ANOVA. This revealed a significant difference between the groups, with [*F*(2, 403) = 235.6, *p* < .001, η^2^ = .54]: the excluded [*M* = 61% correct, *SD* = 22.4, *p* < .001] and classic [*M* = 48% correct, *SD* = 20.4, *p* < .001] DP participants correctly identified fewer celebrity faces in comparison to the controls [*M* = 88% correct, *SD* = 11.3]. In contrast to our sample, the excluded cases were significantly better at identifying celebrities than the classic DP group [*p* < .001]. In summary, it appears that when a larger sample is tested with participants’ familiarity for the faces controlled for, excluded cases are more mildly impaired in familiar face recognition than classic DP participants.

We wanted to replicate excluded cases being impaired at the group level when using the more conservative diagnostic criteria favoured by many labs (i.e., impaired > - 2 SD on the CFMT *and *FFT). Moreover, as our DP sample was comprised of largely females, we wanted to assess whether excluded male cases were impaired on the CFMT, FFT, and upright CFPT^5^ when tested against control males. To do this, we reanalyzed the data made available by Bate et al. (2019a) who tested a large sample of excluded and classical DP cases. These analyses found excluded participants [all *ps* < .001] and excluded males [all *ps* < .013] were impaired on all tasks relative to the controls, although suffered milder deficits than the classical cases [all excluded cases *p*s < .001, excluded males only *ps* < .037].

In summary, excluded DP cases exhibit objective group-level impairments in every aspect of face processing except for the inverted CFPT trials.

## Discussion

Half of all people who believe they have prosopagnosia do not meet commonly used criteria for a diagnosis (Bate et al., 2019a; Burns et al., 2014a; Murray & Bate, 2020), and are largely excluded from research. We wanted to formally assess the proportion of these excluded prosopagnosia cases to those who do meet common criteria and determine whether they have objective deficits as a group. Using one of the conservative approaches commonly favored by many labs (i.e., requiring CFMT impairments > – 2 SDs below the control mean) we failed to diagnose prosopagnosia in 56% of our cases. These excluded cases were indistinguishable in their symptom severity to those who did meet CFMT criteria for a diagnosis. This suggests excluded cases are not *milder* in terms of the regular face processing difficulties that they report suffering from. Moreover, excluded cases exhibited significant impairments in holistic perception that were comparable to classical prosopagnosia cases. While excluded cases’ impairments in face perception and face memory were less severe than classical cases, they still exhibited deficits that were roughly one standard deviation below the control group’s mean scores

As excluded prosopagnosia cases as a group exhibited considerable impairments on the CFMT, CFPT, FFT, and holistic perception, we hope such objective difficulties show researchers the need to include these individuals in our work. Only by doing so will we be able to fully understand the breadth of the prosopagnosia distribution, and more accurately estimate levels of cognitive impairments in this group.

### A new approach for diagnosing developmental prosopagnosia

Developmental prosopagnosia does not currently have any formal diagnostic criteria, nor is it recognized in the DSM-5. Owing to this absence, a handful of researchers and clinicians have proposed cases must meet impairment of > – 2 SDs on two cognitive tasks of face processing (Albonico & Barton, 2019; Barton & Corrow, 2016; Dalrymple & Palermo, 2016). This is because converging evidence convinces researchers and clinicians that an individual is objectively impaired in face recognition (Barton & Corrow, 2016; Dalrymple & Palermo, 2016). Looking at Table 1, we can see that most labs enforce some form of this rule. However, unquestioning adherence to this approach can lead us to counterintuitive conclusions. For example, someone with DP can complain they regularly fail to recognize people close to them, including family members, but will be told that they do not have prosopagnosia because they do not meet single-case criteria on multiple tasks.

We reject this approach to diagnosing prosopagnosia entirely. When we used group-based analyses to increase power, we revealed objective patterns of impairment in such cases. We therefore call for a paradigm shift away from the single case, cognitive task-based methods for diagnosing prosopagnosia. Instead, we outline a plan here for how DP should be diagnosed going forward, and how excluded and traditional DP cases can be included and analyzed without detracting from researchers’ primary findings. It is important authors, manuscript reviewers, and editors do not feel papers are diminished by the inclusion of excluded cases, as ultimately, we all want the same result: the advancement of knowledge. Moreover, our suggestions will move us closer towards formal diagnostic criteria which are effective at diagnosing every potential DP case that clinicians may come across.

First, we recommend the PI20 should be the primary method for diagnosing and including DP cases in our research. The commonly used cut-off of >64 (Shah et al., 2015) detected abnormally high levels of symptoms in all but one case, the latter of which still met criteria when using the two-tailed Crawford’s *t* test approach [*Control PI20 M* = 39.1, *SD* = 8.9, *n* = 48, *Excluded Case’s PI20 score* = 62, *p* = .014]. Moreover, the three control participants who reported regular difficulties in daily life would also have been correctly categorized as suffering from prosopagnosia when using this approach. This suggests to us that impairment identified using the prosopagnosia index questionnaire with a slightly more liberal approach (i.e., Crawford’s *t* test or > 60) than the current > 64 cut-off (Shah et al., 2015), coupled with a self-reported confirmation of prosopagnosia in such cases, will suffice for a diagnosis. Researchers should additionally exclude cases who may have another neurological cause of their problems (e.g., brain damage or schizophrenia). After diagnosing all potential cases through the PI20, we recommend continuing to report the CFMT, CFPT, and FFT scores as is the current norm (Table 1). This will allow researchers to compare the sample’s neuropsychological characteristics to others in the literature.

Given its simplicity and ease of replicability, and similar to other authors (DeGutis et al., 2022), we recommend researchers standardize their categorization of DP cases as mild (i.e., the excluded cases we reported here who scored better than – 2 SDs the CFMT norm) or major (i.e., poorer than > – 2 SD cut-off on the CFMT). While we have previously avoided these terms because the symptom severities are near identical between excluded and classic cases (Fig. 3), we do find that the excluded cases are able to perform better on almost all face processing measures, including the famous faces test when we used a prior dataset (Bate et al., 2019a). As the CFMT is a standardized test that almost all labs are already using, and the cut-off is the most frequently used, it will be easy for researchers to make cross study comparisons due to this universal categorization process.

Some authors have suggested excluding those who fail to score below – 1 SD relative to control means on two cognitive tasks (DeGutis et al., 2022). This approach has been taken from current DSM-5 guidance for diagnosing neurocognitive disorders (Sachdev et al., 2014). However, such exclusions will only serve to perpetuate the issues we summarized in our Introduction. We therefore recommend all researchers use the PI20 to diagnose the condition, as only this approach will allow us to understand the full DP spectrum.

A bonus for using the PI20 as the primary diagnostic tool is that it will be much easier for medical practitioners to diagnose prosopagnosia (Shah, 2016). The PI20 only takes a couple mins to administer and gives an almost unequivocal diagnosis for somebody who believes that they have the condition. By contrast, the commonly used Cambridge Face Memory and Famous Faces Tests together take roughly 20 min and can fail to meaningfully distinguish many prosopagnosia cases from controls when using the single-case approach. In theory, the PI20’s simplicity would mean diagnosing prosopagnosia is much easier for time constrained medics and patients, and will subsequently increase the broader population’s knowledge of the condition due to their exposure to the increased numbers of identified cases. This will hopefully allow prosopagnosia researchers’ work to be more meaningful and impactful on a much more informed and interested society.

### A new approach for researching developmental prosopagnosia

At this point, there are several options available to how prosopagnosia researchers will report their results, i.e., comparisons between controls plus excluded and classical cases on tasks of interest in their papers. We realize some may be concerned that including excluded prosopagnosia cases will reduce their chances of finding significant results. This is because effect sizes will potentially be smaller, given that excluded cases are likely to have milder impairments in other cognitive domains. This is an issue for authors because journals traditionally publish mostly significant findings (Rosenthal, 1979), with researchers’ careers heavily reliant upon such papers (Qiu, 2010; Wang et al., 2019a). By contrast, null results have been commonly ignored, or difficult to publish (Franco et al., 2014; Mervis, 2014; Rosenthal, 1979). Thus, if effect sizes are smaller, it may be harder for researchers to find significant effects in DP, publish in higher impact journals, and have more successful careers^6^.

Given these issues, we recommend researchers perform primary analyses between their classical (i.e., major DP) cases and controls as has historically been the norm. They could then report additional analyses comparing the excluded cases to the controls and classical cases, and subsequent final analyses with both DP groups combined. This means authors do not lose any novelty associated with how their findings can be reported in their paper and its title, e.g., “Major developmental prosopagnosia cases are impaired in Variable X”, but it will allow for additional findings to be reported about excluded cases so that we can start to learn more about this group. Moreover, it will give us estimates of impairment-related effect sizes when the excluded cases are included.

If authors continue to exclude DP cases on whatever basis, then they must explain why this was conceptually important. Given that the distribution of CFMT scores for the excluded and classical DP cases suggests that the former merely inhabit the top end of the normal distribution of the DP group, there seems little reason in our minds to continue excluding them, particularly when they report similar problems as classical DP cases. Obviously, there will be a lag between when the recommendations in the current paper are published to when authors can employ them, as there will be many projects currently being completed without excluded cases. However, it would be helpful if researchers in this position acknowledge that this was the case when publishing their work. Going forward though, researchers should include all potential DP cases in their work.

There currently exists a vast literature that has assessed many aspects of cognitive functioning in DP, including emotion (Biotti & Cook, 2016; Burns et al., 2017c; Dinkelacker et al., 2011; Palermo et al., 2011; Tsantani et al., 2022a, 2022b), object (Geskin & Behrmann, 2018), and word (Burns & Bukach, 2021, 2022; Burns et al., 2017a; Gerlach & Starrfelt, 2022; Rubino et al., 2016; Starrfelt et al. 2018) processing. As almost all papers have tested classical DP cases, we do not know if their conclusions are applicable to those who have been excluded. It will therefore be useful for the field to carry out a series of replication efforts to determine if excluded cases report similarly impaired or spared cognitive processes. These studies could benefit from a registered report format (Chambers, 2013), where research plans are reviewed before being carried out, and accepted by journals irrespective of the conclusions reached.

### Exclusion issues may be present in acquired prosopagnosia too

In contrast to DP, which is characterized by lifelong difficulties with faces, acquired prosopagnosia will typically appear after some form of brain damage, such a stroke or head injury. We have only tested a handful of acquired cases ourselves, and all but one of them have met conservative single-case criteria on tests such as the CFMT. This hints that many of the troubles we have identified with the single-case approach may not be as applicable to acquired prosopagnosia; maybe their deficits are much more severe than those experienced in DP.

However, we should be cautious making this assumption. For example, acquired prosopagnosia cases can score in the neurotypical range on multiple facial identity tasks (Fysh & Ramon, 2022), like what we observe with DP cases. This suggests that the issues we have discussed here may be impacting the acquired prosopagnosia literature too, where cases are excluded from research because they fail to meet conservative criteria on cognitive tasks. Like Eleanor in Fig. 1, they may have had a premorbid test score above the neurotypical mean, but after their stroke, suffered a – 2 SD impairment on this diagnostic measure. They now, alarmingly to the patient, regularly fail to recognize people they should such as their partner, but because they were above average in their premorbid abilities, they will not meet conservative single-case criteria required for a diagnosis^7^. This may occur in clinical practice, where people report problems with faces, but are rejected from assistance because they score in the normal range on diagnostic cognitive tasks, i.e., clinicians do not believe their subjective complaints. Further work will be required to systematically confirm the frequency with which such cases are occurring to determine the extent to which the single-case approach is similarly failing them.

### The PI20 should be used to exclude prosopagnosia cases from neurotypical work

We and others (Bate et al., 2019a; Murray & Bate, 2020) have shown that roughly half of all DP cases will suffer from severe problems when recognizing faces over a short retention period, such as through the CFMT. Roughly 20–25% will have similar difficulties when matching faces (Bate et al., 2019a), and possibly 75% having problems with highly familiar faces (Bate et al., 2019a). In addition, many DP cases have been found to suffer from qualitatively atypical processing of faces. For example, some individuals with DP will exhibit neural (Eimer et al., 2012) and eye movement (Bate et al. 2008) signals and that suggest some form of recognition for faces they have been exposed to, yet will not consciously be able to use this information in order to confirm the person’s familiarity. Moreover, many cases exhibit severe difficulties when processing other forms of social information (Biotti & Cook, 2016; Burns et al., 2017c) and real-world objects with which they have visual expertise with, such as cars and bicycles (Barton et al., 2009; Barton et al., 2019; Burns et al., 2019). Given DP cases’ qualitative abnormalities and severe quantitative deficits in visual processing, we recommend researchers interested in neurotypical visual perception should use the PI20 to exclude potential DP cases from their work. This will ensure DP cases do not unduly influence authors’ data in a way that is difficult to understand.

### Limitations to our new approach?

We should address potential limitations others may see with our new approach. First, some researchers argue that self-report questionnaires like the PI20 do not accurately depict face recognition abilities (Matsuyoshi & Watanabe, 2021; Palermo et al., 2017). This is because the associations between such questionnaires only weakly-to-moderately correlate with performance on cognitive tasks such as the CFMT (Bobak et al., 2019; Estudillo, 2021; Estudillo & Wong, 2021; Livingston & Shah, 2018; Matsuyoshi & Watanabe, 2021; Shah et al., 2015; Palermo et al., 2017; Tsantani et al., 2021; Ventura et al., 2018). Given that experimental tests provide objective data based on experiences designed to imitate those found in the real world (e.g., viewing a face and having to identify it), researchers assume that they must engage actual processes used in day-to-day life. By contrast, self-reported questionnaire data are subjective, not strongly related to performance on these cognitive tasks, and is thus in turn not reflective of objective face recognition abilities.

However, researchers and clinicians rarely question whether these are logical assumptions. For example, it is possible some cognitive tasks may suffer poor ecological validity, where they do not accurately reflect real-world abilities. In this view, questionnaires such as the PI20 may be more effective at providing insights into people’s skills. Indeed, the literature seems to hint that this may at times be correct. For example, young children have been shown to exhibit floor effects (e.g., they are unable to recognize faces) when tested on versions of the CFMT that use children’s faces (Dalrymple & Palermo, 2016). Looking solely at the data, one would conclude all young children have prosopagnosia, but such a hypothesis is easily refuted by watching most children effortlessly recognize their friends and teachers in a school. Similarly, it seems reasonable to assume that these children would not report any difficulties recognizing people they personally know. In this instance, cognitive tests provide little insight into their face recognition abilities.

Cognitive tests’ limitations are also demonstrated in the DP cases we have observed here. If all self-reported DP cases have DP, then their PI20 scores accurately reflect their severe difficulties in daily life. This is because all cases’ symptoms extend multiple SDs beyond control norms. Despite this, cognitive tasks such as the CFMT and CFPT repeatedly failed to capture these problems at the single-case level, i.e., most DP cases score within the normal range. Conversely, super recognizers will often fail to score atypically high on such tasks when assessed individually, but can report unusual examples of excellent real-world recognition (Bate et al., 2019c; Bobak et al., 2016a, b; Ramon et al., 2019). If DP cases and super recognizers are inhabiting extreme ends of the face recognition spectrum in the real world, then we should find this reflected in the single-case analysis of their cognitive task data if these tests have extremely high ecological validity, i.e., all DP cases should be atypically poor, all super recognizers atypically good. However, this patently does not happen in practice (Bate et al., 2019c; Bobak et al., 2016a, b; Ramon et al., 2019). Thus, we need to be cautious in assuming that cognitive tests are providing an excellent reflection of real-world abilities, and that they are superior for diagnosing DP in comparison to questionnaires such as the PI20.

This leads us onto a second problem that may arise from using the PI20 to diagnose DP: how can we validate it if cognitive tests are ineffective at tapping into real-world abilities? While we have argued that the way researchers use such tasks to diagnose DP have serious limitations, please do not misinterpret this as us claiming that they have *no* ecological validity. For example, we have shown close to half of all DP cases will be diagnosed as atypically poor at the single-case level when using the CFMT, and almost a quarter when using the CFPT. Also, we find that the remaining excluded DP cases exhibited impairments when group-based analyses were used to increase power. Thus, even though these tests are imperfect for diagnosing every individual person with DP, they must tap into *some* of the issues that they are experiencing in the real world. We therefore believe such tasks can be used to validate the PI20, despite the serious limitations which force us to reject them as diagnostic tools.

A final issue some may raise with the PI20 is that it has yet to undergo further work to meet common gold standards used in scale development. For example, it is recommended that a panel of experts independent of the scale developers evaluate the questions to be included (Boateng et al., 2018). This will help remove redundant, imprecise, and inaccurate items, and allow any missed questions and factors to be highlighted (Augustine et al., 2012; Boateng et al., 2018). Moreover, while the PI20 developers used an exploratory factor analysis to show the scale reflects a single factor (i.e., prosopagnosia), they did not perform confirmatory factor analyses in a longitudinal sample (e.g., months later) or in an independent sample as is recommended (Boateng et al., 2018). Nor did they try to develop their scale further to see if there might be multiple factors that could reflect different aspects of face processing. When a group of independent experts critiqued and adapted the PI20’s items (Bobak et al., 2019), they found that there may be two subcomponents in their face processing questionnaire. This hints that there may be some benefits to be had from further refinement of the PI20.

Despite this, the PI20 seems extremely effective at fulfilling its original purpose: diagnosing DP. For example, using a PI20 cut-off of ≥ 60 here resulted in all people who believe they have prosopagnosia diagnosed with the condition, and almost all neurotypicals correctly classified as not suffering from prosopagnosia. If all people who believe that they have prosopagnosia do indeed suffer from it, then the PI20 has excellent diagnostic utility, particularly when paired with a self-reported complaint that the individual is suffering from regular problems in daily life. This means that despite there being scope for the PI20 to undergo further development, it is the best diagnostic tool researchers and clinicians have available.

## Conclusions

We have demonstrated that excluded DP cases are impaired in almost every aspect of face processing as assessed by commonly used neuropsychological tests. In some cases, they suffered comparable difficulties (e.g., prosopagnosia symptoms, holistic perception) as classical cases, suggesting key features shared by both subgroups of this disorder. We have set out a new plan for researchers to diagnose and research prosopagnosia, one which will allow them to include all individuals who believe that they have this condition. Moreover, our recommended approach will avoid any diminishment of researchers’ findings than if they had solely tested classical DP cases. We anticipate such an approach will allow for much more accurate estimates of treatment efficacy, cognitive impairments, and prevalence of this condition.

## Data Availability

All data required to replicate our results is available on the Open Science Framework (https://osf.io/tx5av/?view_only=a69e476ec5614833a4075eaff01e0d4e). As we do not own the copyright for the CFMT, CFPT and PI20, we do not provide materials for these tasks, but we have made our Famous Faces Test script and images publicly available on the above link. The PI20 is freely available in the original paper (Shah et al. 2015). The CFMT and CFPT were kindly provided by Brad Duchaine.

## Footnotes

1 A remarkably similar, albeit converse, problem is apparent for super recognisers (Russell et al. 2009): individuals with exceptional face processing abilities who can, for example, recognize a person’s face even if they have only briefly met many years previously. Many of these individuals can fail to score atypically well at the single-case level on face processing tasks, although do as a group appear superior when compared to the general population (Bate et al. 2019c; Bobak et al. 2016a, b; Ramon et al. 2019). This hints that face processing tasks may be failing to accurately depict real-world abilities at extreme ends of the face recognition continuum, i.e., prosopagnosia and super recognition.

2 This exact argument was proposed in a recent paper to highlight the risks associated with low power when determining impairment in single cases (McIntosh & Rittmo, 2021). We have adapted it here for developmental prosopagnosia, but it is relevant for other neurodevelopmental and neuropsychological conditions. We urge researchers to read this paper as it convincingly outlines the power limitations of the single-case approach in much greater detail than we provide here.

3 These discussions are common in other neurodevelopmental disorders, such as autism (Abu-Akel et al. 2019; Chown & Leatherland, 2020; Happé & Firth, 2020, 2021; Elton et al., 2016; Kim et al. 2019; Tang et al. 2020) and dyslexia (Cilibrasi & Tsimpli, 2020; Wagner et al. 2020). Both conditions have garnered a great deal more research into them than prosopagnosia (Google Scholar’s number of papers with term in title: ‘autism’, 213000; ‘dyslexia’, 24800; ‘developmental prosopagnosia’, 308), and yet, authors are still uncertain which hypothesis best fits the data. Notably, there appear to be problems when using the categorical approach to diagnose dyslexia, with cases seemingly being missed (Cilibrasi & Tsimpli, 2020) like what we observe in DP.

4 We have until this point avoided referring to these individuals as suffering from *mild* prosopagnosia. While they do not meet single-case criteria on tests, we do not know yet if their problems with faces are actually milder when compared to those who do meet criteria. If excluded prosopagnosia cases are indistinguishable in their symptom severity from those who satisfy criteria, then is it fair to call their condition mild?

5 We use these test scores as they were the only data available to analyze.

6 We should note that potentially smaller effect sizes will be offset by increased numbers of DP cases available to test.

7 This is a common issue in dementia, where highly intelligent individuals can detect failings in their cognitive abilities as dementia onsets, but owing to their high-performing premorbid state, they are rejected from a diagnosis because they are not sufficiently impaired (Nelson & O’Connor, 2008).
